# Utilization of hypoxia-derived gene signatures to predict clinical outcomes and immune checkpoint blockade therapy responses in prostate cancer

**DOI:** 10.3389/fgene.2022.922074

**Published:** 2022-08-12

**Authors:** Minhua Chen, Zhang Chen, Zongbin Lin, Xiang Ding, Tianyu Liang

**Affiliations:** ^1^ Emergency & Intensive Care Unit Center, Department of Intensive Care Unit, Zhejiang Provincial People’s Hospital, Affiliated People’s Hospital, Hangzhou Medical College, Hangzhou, China; ^2^ Department of Urology, The First Affiliated Hospital of Soochow University, Suzhou, China

**Keywords:** prostate cancer, hypoxia, immune checkpoint blockade, prognostic, outcome

## Abstract

**Background:** Increasing evidences show a clinical significance in the interaction between hypoxia and prostate cancer. However, reliable prognostic signatures based on hypoxia have not been established yet.

**Methods:** We screened hypoxia-related gene modules by weighted gene co-expression network analysis (WGCNA) and established a hypoxia-related prognostic risk score (HPRS) model by univariate Cox and LASSO-Cox analyses. In addition, enriched pathways, genomic mutations, and tumor-infiltrating immune cells in HPRS subgroups were analyzed and compared. HPRS was also estimated to predict immune checkpoint blockade (ICB) therapy response.

**Results:** A hypoxia-related 22-gene prognostic model was established. Furthermore, three independent validation cohorts showed moderate performance in predicting biochemical recurrence-free (BCR-free) survival. HPRS could be a useful tool in selecting patients who can benefit from ICB therapy. The CIBERSORT results in our study demonstrated that hypoxia might act on multiple T cells, activated NK cells, and macrophages M1 in various ways, suggesting that hypoxia might exert its anti-tumor effects by suppressing T cells and NK cells.

**Conclusion:** Hypoxia plays an important role in the progression of prostate cancer. The hypoxia-derived signatures are promising biomarkers to predict biochemical recurrence-free survival and ICB therapy responses in patients with prostate cancer.

## Introduction

Prostate cancer (PC) is the third common cancer among all cancers and the second cancer among men worldwide (Sung et al., 2021)**.** In 2020, 1,414,259 people suffered from PC and 375,304 patients died of PC globally (Sung et al., 2021). Existing evidence suggests that the proliferation and progression of PC are strongly dependent on androgen receptor (AR) signaling (Tan et al., 2015; Crawford et al., 2018). Although androgen deprivation therapy (ADT) is the standard clinical therapy and most patients initially respond to hormone therapy, they eventually relapse and progress to biochemical recurrence (BCR). Therefore, it is urgent to find methods that can better predict tumor prognosis and therapy response than the existing ones.

One of the main reasons that cancers are difficult to treat is that cancer cells constantly adapt to the adverse environment in which they live. Hypoxia is one such adverse environment, which can impair the function of the tumor. On the contrary, hypoxia drives the tumor to produce more malignant characteristic behavior (Chae et al., 2016). We presumed that perhaps hypoxia-related features could be used to predict tumor prognosis and drug response. However, hypoxia gene signatures were successfully obtained in multiple tumors including clear cell renal cell carcinoma, hepatocellular carcinoma, bladder cancer, lung adenocarcinoma, glioma, and breast cancer, and these gene signatures not only independently predicted prognosis in lung adenocarcinoma but also predicted therapeutic resistance (Gui et al., 2021; Mo et al., 2021) (Gong et al., 2020; Lin et al., 2020; Sun et al., 2020; Cao et al., 2021). Recently, immunotherapy has emerged as a new type of therapy that has brought hope to cancer patients, but there are also many patients who do not respond to this treatment. Hypoxia also affects the immune system through multiple pathways, such as induction of transcription factors or target genes to suppress T-cell proliferation and induction of mitochondrial stress that drives T-cell exhaustion (Scharping et al., 2021) (Barsoum et al., 2014).

In this study, we performed hypoxia assessment on samples in the training dataset, screened out hypoxia-related genes using weighted gene co-expression network analysis (WGCNA), and finally established a 22-gene signature and validated it in multiple independent datasets. As hypoxia affects the immune system, we also evaluated risk models and immune checkpoint blockade (ICB) therapy.

## Materials and methods

### Data acquisition

Public gene-expression data and full clinical annotation were searched in the Gene Expression Omnibus (GEO; https://www.ncbi.nlm.nih.gov/gds/), The Cancer Genome Atlas (TCGA; https://portal.gdc.cancer.gov/), and the Array Expression (https://www.ebi.ac.uk/arrayexpress/) databases. The procedure used for dataset selection in the GEO database was as follows. The following search parameters were used: [“prostatic neoplasms” (MeSH Terms) OR prostate cancer (All Fields)] AND “Homo sapiens” (porgn) AND [“gse” (Filter) AND “Expression profiling by array” (Filter)]. In the initial search, 924 items were recognized. The eligible criteria included: 1) owning BCR time information and 2) at least 50 PC patients. We removed the datasets that do not meet the criteria by checking them one by one carefully and gathered three patient cohorts from GEO. We searched the Array Expression (https://www.ebi.ac.uk/arrayexpress/) and found a dataset that met the criteria. In total, we gathered five patient cohorts for this study: TCGA-PRAD, GSE116918 (Jain et al., 2018), GSE46602 (Mortensen et al., 2015), GSE70770 (Ross-Adams et al., 2015), and E-MTAB-6128. Three GEO datasets are processed expression matrices downloaded from GEO. The CEL files from E-MTAB-6128 were downloaded and normalized using a robust multichip average (RMA) algorithm (Irizarry et al., 2003). All microarray data included in our study were log2-transformed. The TCGA-PRAD RNA-seq data and clinical data were downloaded using the “TCGAbiolinks” package in R ((Colaprico et al., 2016)). The RNA-seq data were converted to transcripts per million (TPM) after removing duplicated genes and zero expression genes. The dataset GSE116918 was used as the training set because it is an independent microarray dataset with an appropriate sample size (Jain et al., 2018). Moreover, another three datasets from different response platforms were used as three independent validation sets including GSE46602, GSE70770, E-MTAB-6128, and TCGA-PRAD. TCGA-PRAD somatic mutation data were downloaded from TCGA using the package TCGAbiolinks in R. Somatic mutation data were analyzed using the R package “maftools” (M ayakonda et al., 2018). In order to analyze the relationship between 22 gene signatures and immunotherapy, we found an ICB therapy dataset (IMvigor210) with 299 tumor samples and survival information. Raw transcriptome and clinical data were retrieved from the IMvigor210 dataset (http://research-pub.gene.com/IMvigor210CoreBiologies) using the R package “IMvigor210CoreBiologies” (Mariathasan et al., 2018). The hypoxia-associated gene sets (HALLMARK_HYPOXIA and HARRIS_HYPOXIA) were obtained from the MSigDB (http://www.gsea-msigdb.org/gsea/msigdb/index.jsp).

### Screening of hypoxia-related genes and establishment of a risk model

The levels of hypoxia in each sample from GSE116918 were quantified using a single-sample gene set enrichment analysis (ssGSEA) algorithm based on hallmark gene sets and chemical and genetic perturbation sets (Hänzelmann et al., 2013). To find modules highly correlated with hypoxia, WGCNA was performed using the “WGCNA” R package (Langfelder and Horvath, 2012) and carried out on the top 50% most variable genes. We calculated that the most β-value is 3, and the minimum number of genes in the module is set to 50. After screening the hypoxia-related modules, univariate Cox regression analysis was performed on the genes in the modules. The genes with *p* < 0.01 were defined to be related to BCR time. Then, we used the R software package “glmnet” to integrate BCR time, BCR status, and gene expression data for regression analysis using the LASSO-Cox method. In addition, we also set up 10-fold cross-validation to obtain the optimal model. Finally, a hypoxia-related prognostic risk score (HPRS) was constructed: HPRS = ∑(C×EXP), where EXP is the expression value of the gene and C is the regression coefficient for the corresponding gene in the LASSO-Cox model.

### Hypoxia signature validation

We used the R package “maxstat” to calculate the optimal cutoff value of HPRS, setting the minimum number of grouped samples to be greater than 25% and the maximum number of samples to be grouped less than 75%. Based on this, the patients were divided into two groups, high-risk and low-risk, and the prognostic difference between the two groups was further analyzed using the Kaplan–Meier (KM) method. The log-rank test method was used to evaluate the significance of the prognostic difference between samples in different groups. Also, the receiver operating characteristic (ROC) curve is also used to evaluate the prediction performance of the hypoxia-related signature in the training set and the validation set. We used the R package “rms” to integrate data on BCR time, BCR status, and other clinical characteristics and constructed a nomogram using the Cox method and assessed the prognostic significance of these characteristics in GSE116918.

### Estimation of immune cells

The CIBERSORT algorithm (https://cibersortx.stanford.edu/) with the B-mode of batch correction mode, relative mode, and 1,000 permutations was used to estimate the fraction of 22 immune cell types in PC samples (Newman et al., 2019). The Wilcoxon test was used to look for significantly differential immune cells between high and low HPRS.

### Gene enrichment analysis and GSEA

To illustrate the functional annotations of target gene set, Kyoto Encyclopedia of Genes and Genomes (KEGG) pathway enrichment analyses were performed using the KOBAS 3.0 online database (Bu et al., 2021). Pathways with adjusted *p* < 0.05 were considered significant. We downloaded GSEA software (version 4.3) from the GSEA website (http://software.broadinstitute.org/gsea/index.jsp). We downloaded the latest Kyoto Encyclopedia of Genes and Genomes (KEGG) pathway data using the R package “KEGGREST.” The prepared KEGG gene sets were used as the reference set. We grouped samples according to HPRS as the phenotypic input file. NOM *p*-value < 0.05 was considered statistically significant.

### Additional bioinformatic and statistical analyses

The violin plot is used to compare the differences between two groups with the Wilcoxon test. In order to test the efficacy of HPRS in predicting ICB therapy and avoid overfitting of the predicting model, the ICB therapy was divided two groups on the basis of 8:2, the one in front was named as the train cohort and the one in back was named as the test cohort. Support vector machines (SVMs) are a very good machine algorithm that can be used to distinguish between two variable outcomes. So, we used SVM for the evaluation of HPRS for predicting ICB therapy response. All of the aforementioned analyses were performed using R software (version 4.0.2, http://www.rproject.org). Statistical significance was set at *p* < 0.05.

### Gene expression verification

We analyzed the RNA expression differences of 22 genes in the Gene Expression Profiling Interactive Analysis (GEPIA) database. In addition, we collected 20 pairs of PC samples and adjacent normal samples in our hospital to analyze the RNA expression differences of these genes using RT-PCR, all of which were approved by the patient’s informed consent and the Ethics Committee of Zhejiang Provincial People’ Hospital. TRIzol (Thermo Fisher, United States) was used to extract the total RNA in the sample, and the reverse transcription kit, RR047A kit (Takara, Japan), was used to convert it into cDNA. Finally, the RR820A kit (Takara, Japan) was used to perform RT-PCR analysis on the 7900HT system (Thermo Fisher, United States), and the *ACTB* gene was used as the internal reference gene to calculate the expression of hub genes for each pair of tissues.

## Result

### Estimation of hypoxia level and characteristics of different hypoxia levels

**FIGURE 1:**
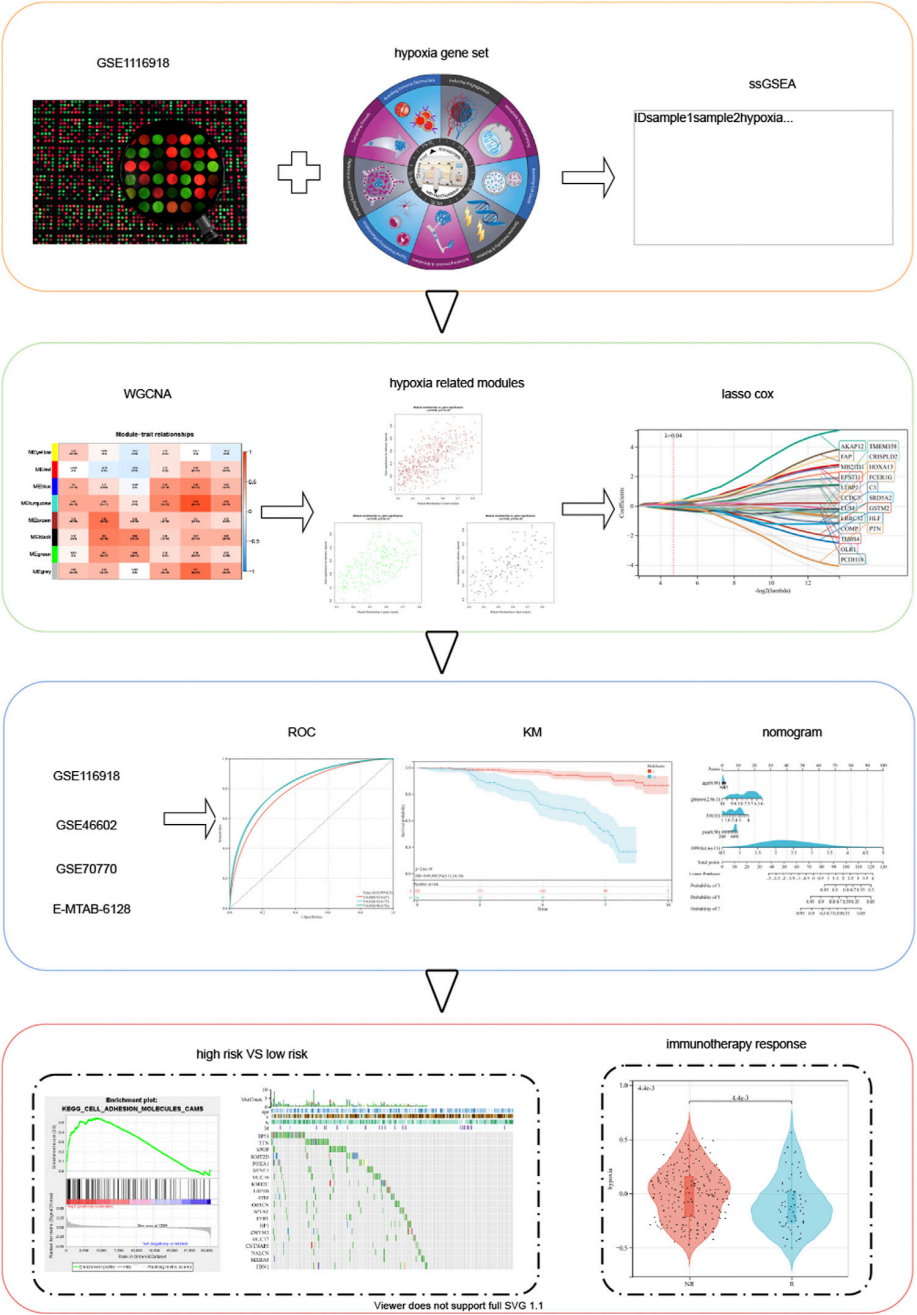
Schematic diagram of the study design.

**FIGURE 2:**
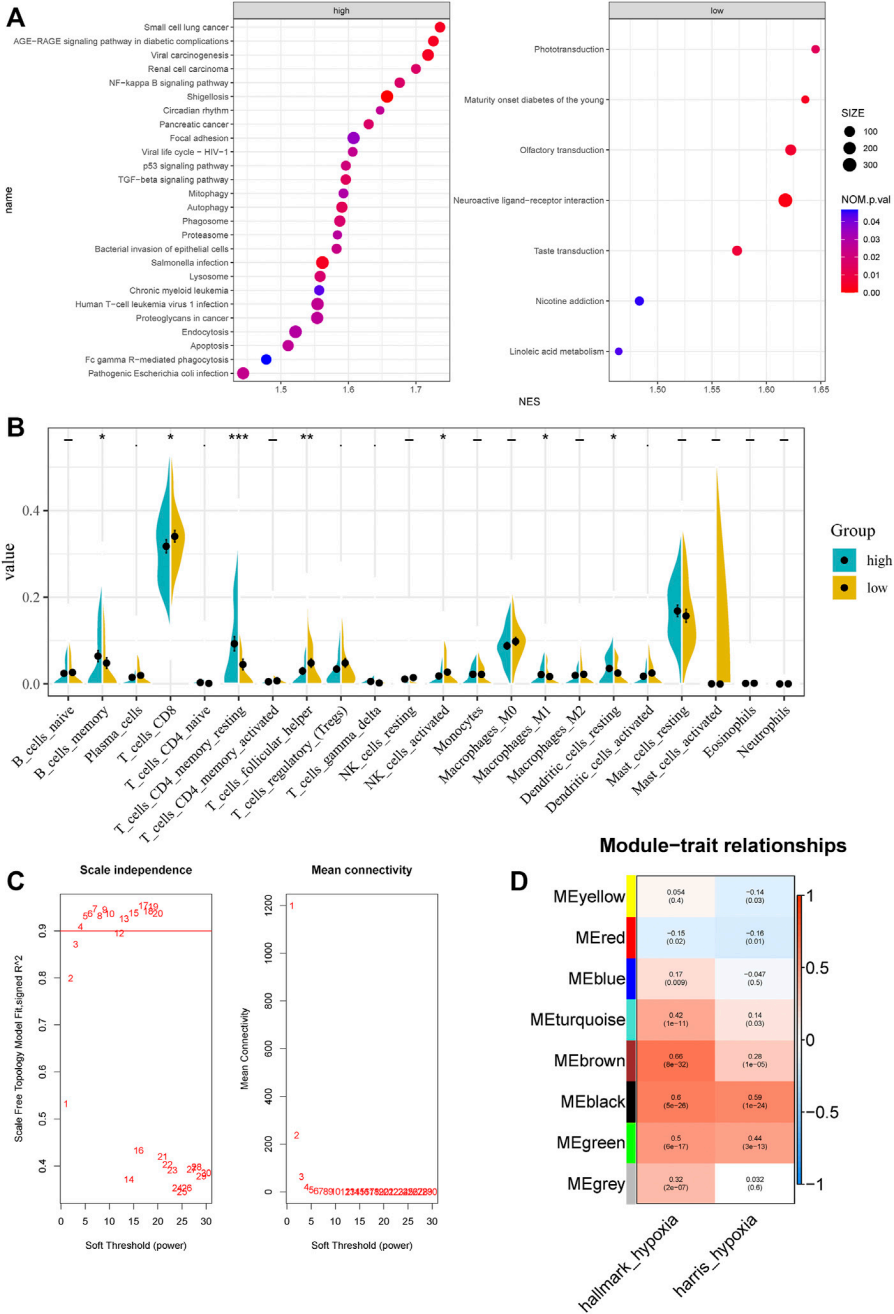
Characteristic between different hypoxia levels and weighted gene co-expression network analysis. **(A)** GSEA analysis of different hypoxia levels. **(B)** CIBERSORT results of different hypoxia levels. **(C)** Analysis of the scale‐free fit index and the mean connectivity for various soft‐thresholding powers. **(D)** Correlation of module eigengenes with two hypoxia scores.

The procedures used in this study are summarized in Figure 1. Based on ssGSEA scores of hallmark hypoxia, we divided patients in the GSE116918 cohort into high and low groups by the median score. GSEA results indicated that the high hypoxia score group was mainly enriched in multiple viral and bacterial infection-related pathways, such as pathogenic *Escherichia coli* infection, *Salmonella* infection, bacterial invasion of epithelial cells, viral life cycle—HIV-1, shigellosis and viral carcinogenesis, indicating that hypoxia correlates with malignant behavior of tumors (Figure 2A). The low hypoxia score group was mainly enriched in linoleic acid metabolism, nicotine addiction, taste transduction, neuroactive ligand–receptor interaction, olfactory transduction, maturity onset diabetes of the young, and phototransduction (Figure 2A). We performed CIBERSORT, and the results indicated that there were significant differences in a variety of immune cells, including CD8^+^ T cells, CD4^+^ memory resting T cells, follicular helper T cells, activated NK cells, M1 macrophages, resting dendritic cells, and activated dendritic cells (Figure 2B).

### Detection of gene co-expression modules correlated with hypoxia

Top 50% most variable genes were used for WGCNA. The soft threshold = 3 was selected to construct a scale-free network (Figure 2C). A total of 7 modules were identified after setting the minimum cluster size at 50 (Figure 2D). Among these modules, the green module (*r* = 0.5, *P* = 6e−17), the black module (*r* = 0.6, *P* = 5e−26), and the brown module (*r* = 0.66, *P* = 8e−32) exhibited the highest correlation with the hypoxia score and was considered the “hypoxia-related module.” Finally, we obtained 831 hypoxia-related genes and found that they are significantly enriched in cancer-related pathways, such as pathways in cancer, PI3K-Akt signaling pathway, phagosome, transcriptional mis-regulation in cancer, and proteoglycans in cancer.

### Development of HPRS in the train set

**FIGURE 3:**
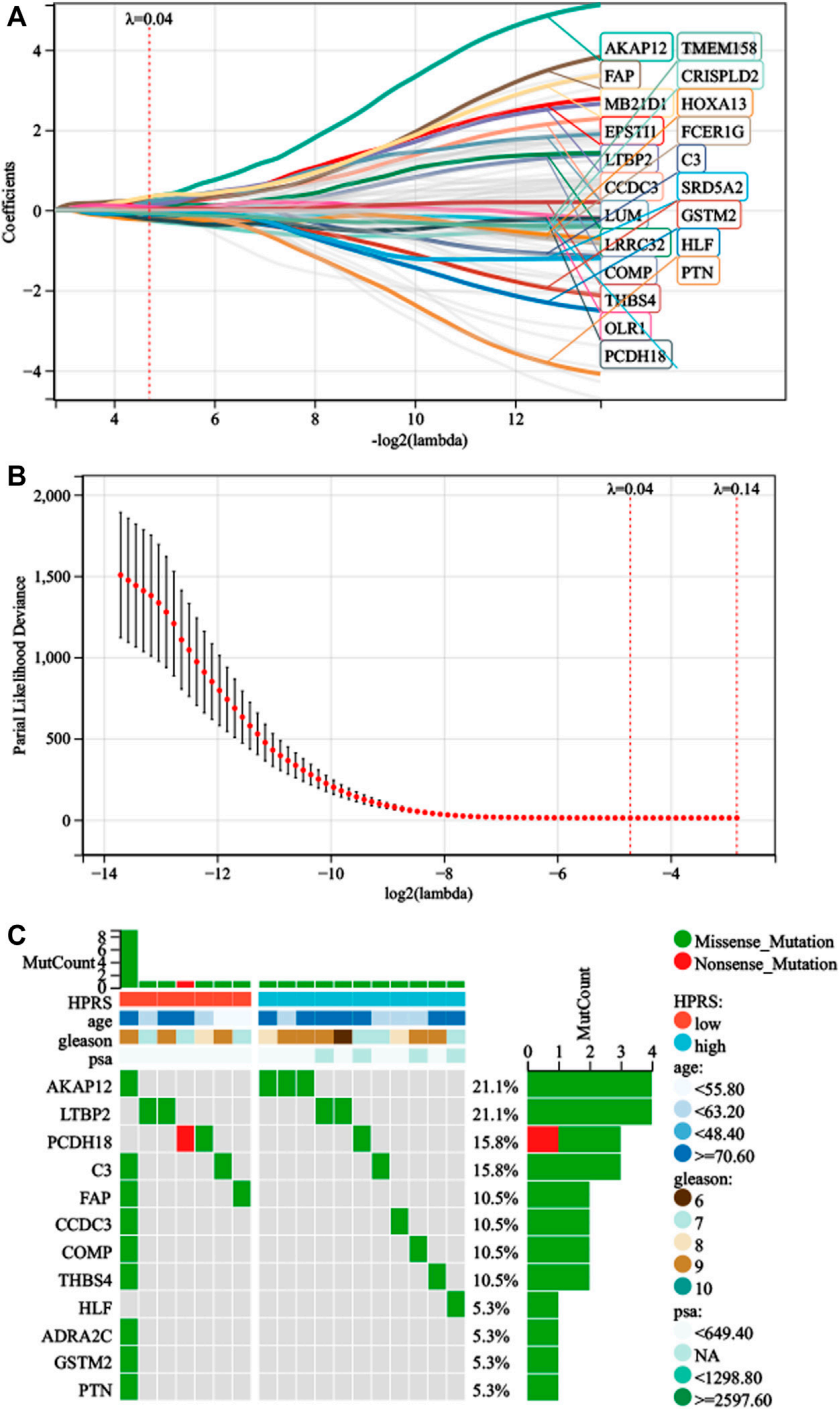
Development of HPRS in the train set. **(A)** Trajectory of each prognosis-related candidate gene’s coefficient was observed in the LASSO coefficient profiles with the changing of the lambda in LASSO algorithm. **(B)** After the 10-fold cross-validation, a confidence interval was obtained for partial likelihood deviance as the lambda changed. **(C)** Mutation status was analyzed of 22-gene signature in the TCGA-PRAD.

First, we performed univariate Cox regression on the genes of the three modules and found 91 genes with *p* < 0.01. Subsequently, LASSO-Cox algorithm was used to identify the most robust prognostic genes. The optimal λ value of 0.04 was selected (Figures 3A,B). Finally, the risk scores of each PC patient were measured using the following method: HPRS = −0.0299179376392757*ADRA2C + 0.320877677041365*AKAP12 + 0.157921193186689*C3 + 0.133671046325701*CCDC3 + 0.0485939928513679*COMP−0.263511901072997*CRISPLD2 + 0.202066976480225*EPSTI1 + 0.210803303885677*FAP + 0.0190836207797853*FCER1G-0.0275655761387271*GSTM2-0.124475388639999*HLF−0.235913610478483*HOXA13 + 0.0633028925180767*LRRC32 + 0.135931506683704*LTBP2 + 0.203447730218895*LUM + 0.334111004412106*MB21D1 + 0.0631829402327768*OLR1−0.227346268601104*PCDH18–0.0345544068599357*PTN−0.0618838003001662*SRD5A2 + 0.0052758696780799*THBS4−0.0883863753799873*TMEM158. The mutation data of these 22 genes in the TCGA-PRAD dataset are shown in Figure 3C. The 22 genes were significantly correlated with the phagosome pathway (corrected *p* = 7.96e−05).

### Validation of HPRS

**FIGURE 4:**
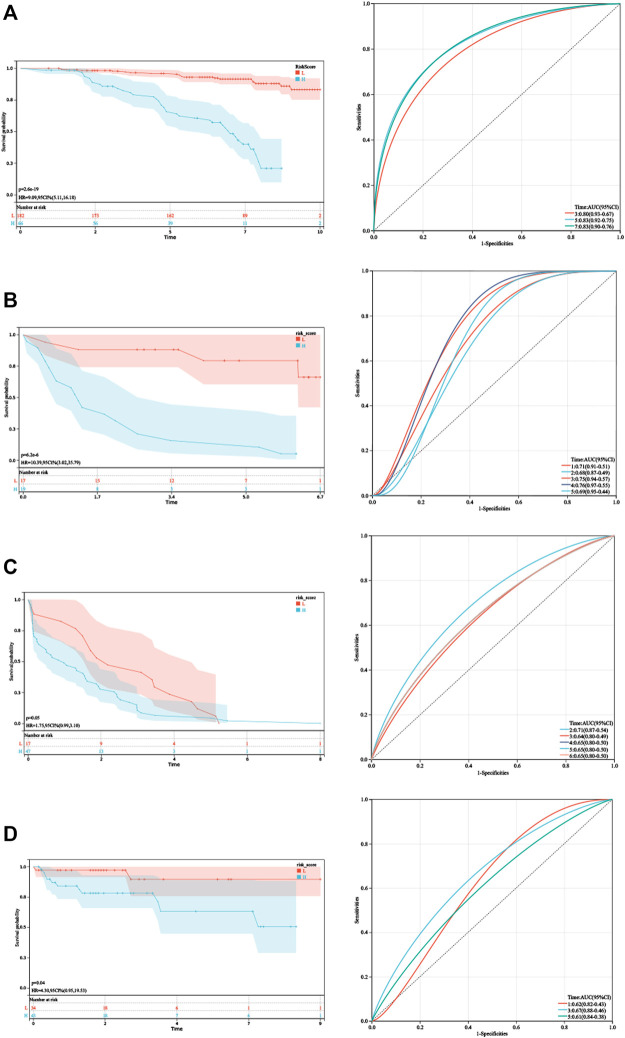
Validation of HPRS. **(A)** Kaplan–Meier analysis and ROC analysis of the hypoxia-related score based on GSE116918. **(B)** Kaplan–Meier analysis and ROC analysis of the hypoxia-related score based on GSE46602. **(C)** Kaplan–Meier analysis and ROC analysis of the hypoxia-related score based on GSE70770. **(D)** Kaplan–Meier analysis and ROC analysis of the hypoxia-related score based on E-MTAB-6128.

As shown in Figure 4, Kaplan–Meier analysis demonstrated that patients with higher HPRS exhibited BCR-free survival in the training set (HR = 9.09, 95% CI = 5.11–16.18, *p* = 2.6e-19). Then, the prognostic value of HPRS was validated in four independent cohorts (GSE46602: HR = 10.39, 95% CI = 3.02–35.79, *p* = 6.2e-6; GSE70770: HR = 1.75, 95% CI = 0.99–3.10, *p* = 0.05; and E-MTAB-6128: HR = 4.30, 95% CI = 0.95–19.53, *p* = 0.04; Figure 4). To evaluate the predictive efficiency of the hypoxia signature, we performed a ROC curve utilizing the data from the train and validation cohorts. The area under the ROC curve (AUC) is shown in Figure 4. HPRS has excellent predictive performance in the train set and also has moderate prognostic ability in the validation datasets (Figure 4). Patients with high HPRS appear to have more BCR (Figure 4).

### HPRS could be utilized as an independent prognostic factor in PC

**FIGURE 5:**
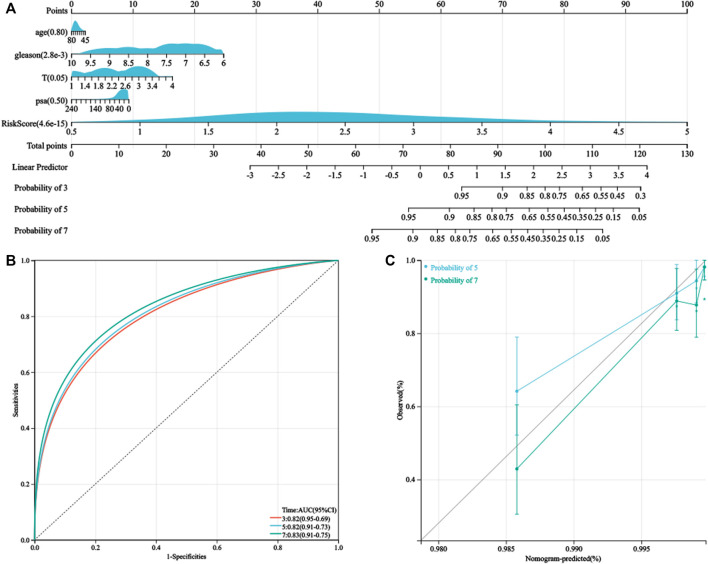
Nomogram was generated to improve risk stratification and estimate survival probability. **(A)** Comprehensive nomogram for predicting probabilities of PC patients with 3-, 5-, and 7-year BCR-free survival in GSE116918 dataset. **(B)** ROC analyses of 3-, 5-, and 7-year BCR-free survival for this nomogram. **(C)** Calibration plots for predicting PC patients with 5- and 7-year BCR-free survival in GSE116918.

As the HPRS was significantly correlated with high malignancy, we sought to determine whether the HPRS was a clinically independent prognostic factor for PC patients through multivariate Cox regression analysis. The HPRS, together with other clinical features, including age, Gleason score, pathological T stage, and prostate-specific antigen (PSA), were enrolled as covariates to perform the analysis. The results demonstrated that HPRS, Gleason score, and pathological T stage were independent factors that could be utilized to predict the prognosis of PC patients. By combining the aforementioned prognostic factors, we constructed a nomogram that served as a clinically relevant quantitative method by which clinicians could predict mortality in PC patients (Figure 5). Each patient would be assigned a total of points by adding points for each prognostic parameter. The higher the total score, the worse the patient’s prognosis. The overall C-index of the model was 0.80, with a 95% CI of 0.74–0.87 and a *p* value of 1.4e-20. The capacity of the nomogram to distinguish survival was tested using AUC values (Figure 5). In the calibration analysis, the prediction lines of the nomogram for 5- and 7-year survival probability were extremely close to the ideal performance (45-degree line) (Figure 5), indicating a high accuracy of the nomogram.

### Comprehensive analyses of enriched pathways and genomic alterations between different risk groups

**FIGURE 6:**
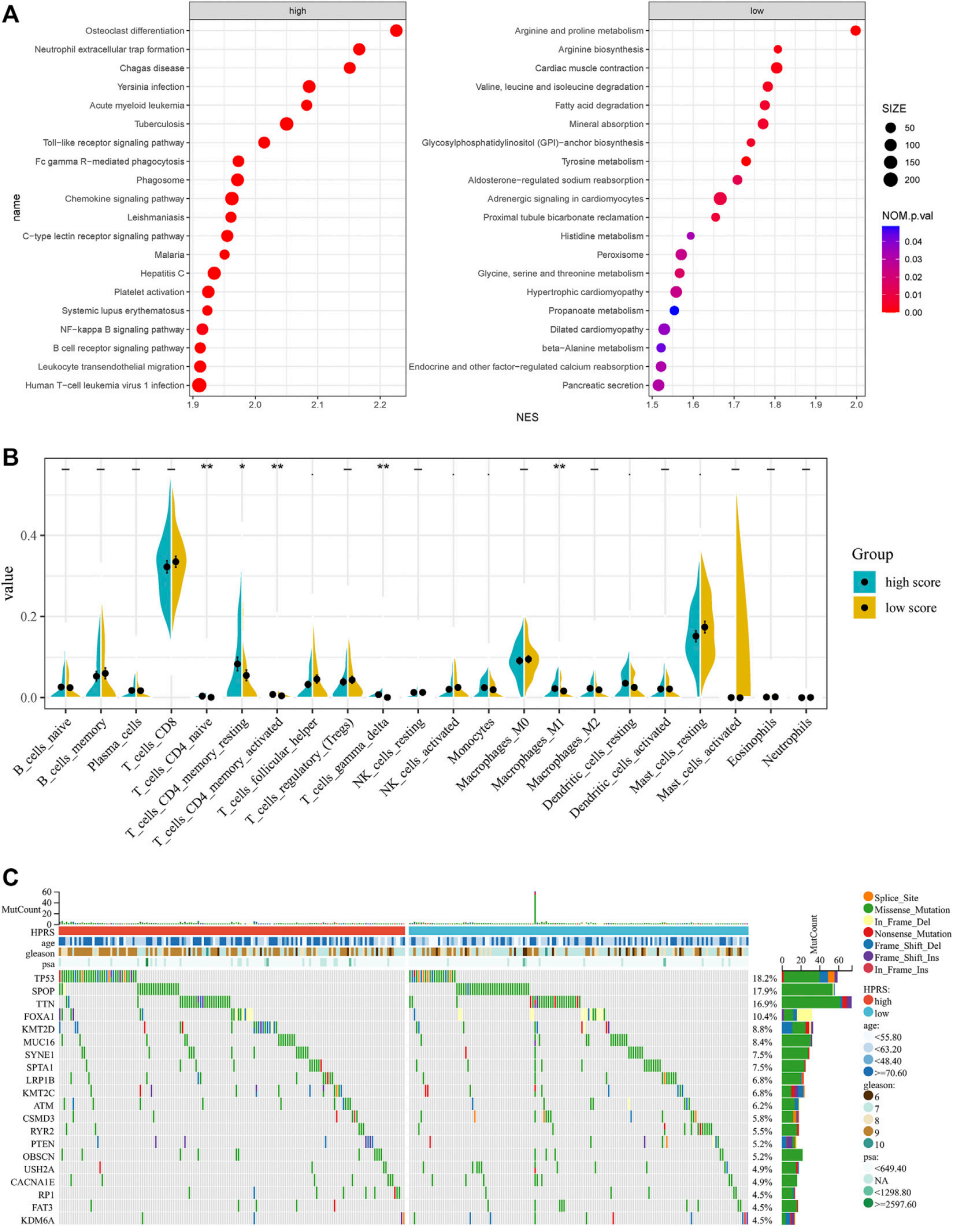
Comprehensive analyses of enriched pathways and genomic alterations between different risk groups. **(A)** GSEA analysis of high- and low-HPRS groups (top 20 pathways). **(B)** CIBERSORT result of high- and low-HPRS groups. **(C)** Top 20 most frequently mutated genes were illustrated in TCGA-PRAD.

To further verify the activation of related signaling pathways in the different hypoxia risk groups, we performed GSEA in the GSE116918 cohort. The results showed that the high-risk group was mainly enriched in immune cell regulation-related pathways, including human T-cell leukemia virus 1 infection, leukocyte trans-endothelial migration, B-cell receptor signaling pathway, and neutrophil extracellular trap formation (Figure 6A). The low-risk group was mainly enriched in metabolism-related pathways, including beta-alanine metabolism, propanoate metabolism, glycine, serine, and threonine metabolism, histidine metabolism, tyrosine metabolism, fatty acid degradation, valine, leucine, and isoleucine degradation, arginine biosynthesis, and arginine and proline metabolism (Figure 6A).

In addition, the low-risk group was also significantly enriched in the prostate cancer pathway (Figure 6A).

Accumulating evidence suggests that hypoxia is an important feature of cancers that can modulate the cancer’s immune response. Using the CIBERSORT method, we estimated differences in the immune infiltration of 22 immune cell types between low- and high-risk PC patients in the GSE116918 cohort. We found that CD4^+^ naive T cells, CD4^+^ memory resting T cells, activated CD4^+^ memory T cells, follicular helper T cells, gamma delta T cells, activated NK cells, monocytes, M1 macrophages, resting dendritic cells, and resting mast cells differed between the two groups (Figure 6B).

To investigate whether there was evidence of differences at the genomic level between low- and high-risk PC patients, we investigated the distribution differences of somatic alterations in the TCGA-PRAD cohort using the R package “maftools.” Waterfall plots integrated with 20 highly variant mutant genes were utilized to show the mutation landscape. As shown in Figure 6C, among the top 20 mutated genes, the high-risk group appeared to have a higher mutation rate relative to the low-risk group.

### Verification of 22 genes in this model

**FIGURE 7:**
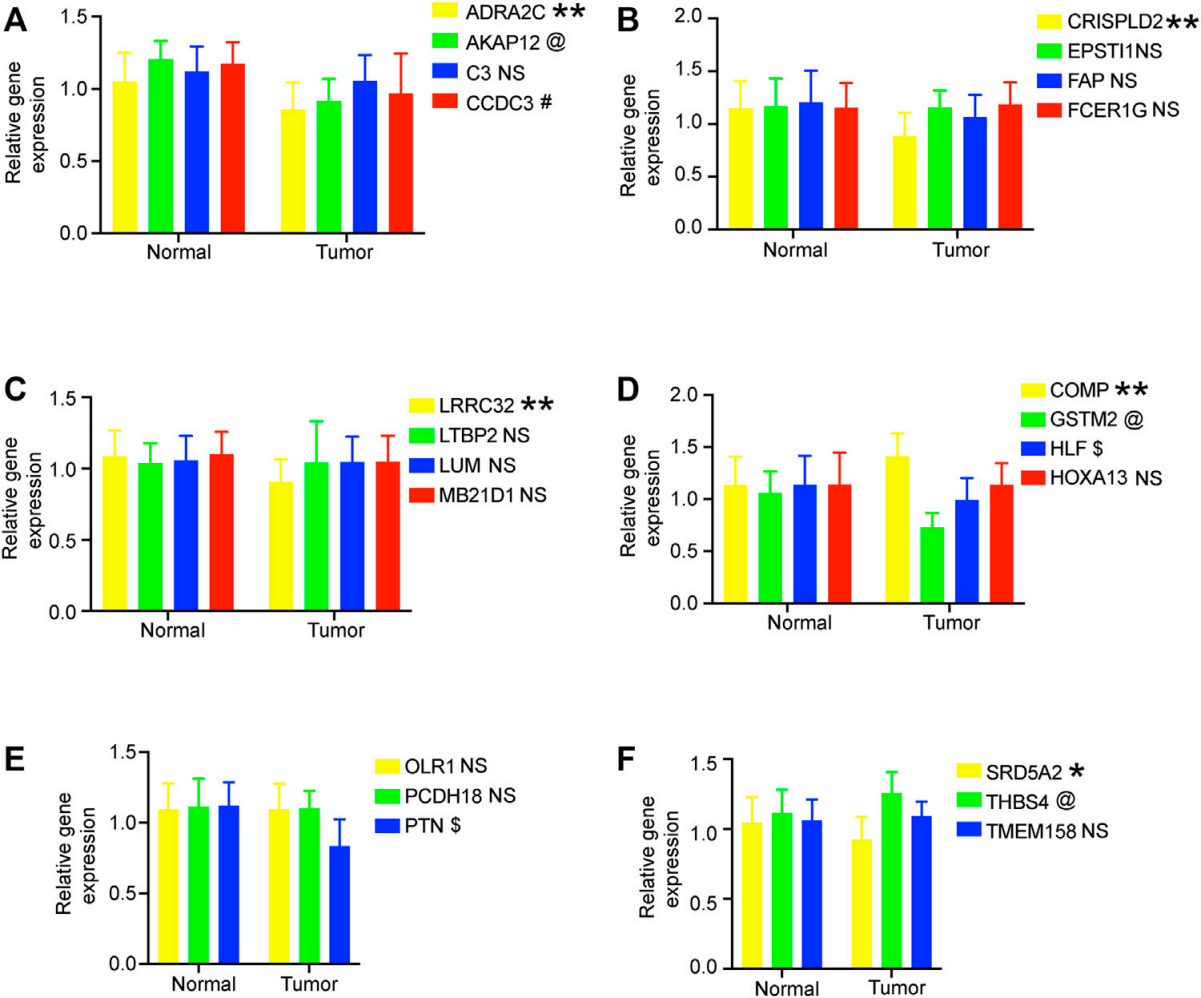
RT-PCR verified the expression of genes in 20 pairs of prostate cancer clinical samples. **(A–F)** Relative gene expression level in the normal group and tumor group. All data are displayed as means ± SD; mean values for the normal group were normalized to 1.0; NS, no significant difference vs. normal group; **p < 0.01 and *p < 0.05 vs. normal group. @*p < 0.05 vs. normal group. ^#^*p < 0.05 vs. normal group. $*p < 0.05 vs. normal group.

We verified the expression levels of 22 genes. In the GEPIA database, we found that 11 genes were differentially expressed in tumor tissues and adjacent tissues, of which 9 genes were downregulated and 2 genes were upregulated in PC. In the 20 pairs of clinical samples we collected, RT-PCR results showed that the expression of *ADRA2C, AKAP12, CCDC3, CRISPLD2, GSTM2, HLF, LRRC32, PTN, and SRD5A2* were reduced in PC. *COMP and THBS4* were overexpressed in PC (Figure 7). These results are consistent with our analysis.

### HPRS predicted ICB therapeutic response

**FIGURE 8:**
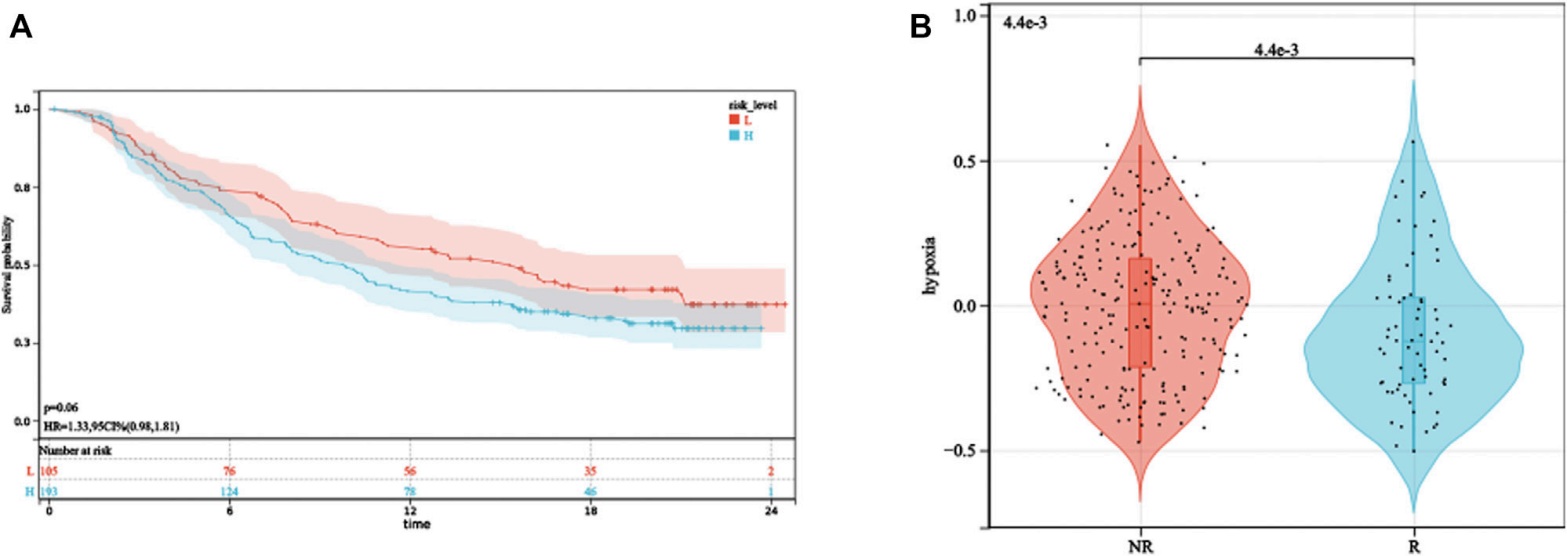
HPRS predicts ICB therapeutic response. **(A)** Kaplan–Meier analysis of HPRS in the IMvigor210 dataset. **(B)** Differences in hypoxia levels between ICB therapy-response and non-response groups.

The aforementioned analysis of tumor-infiltrating immune cells showed that multiple T cells were significantly different between high- and low-HPRS groups, implying that HPRS may be predictive of ICB therapeutic response. As there is no immunotherapy cohort for prostate cancer, we estimated the performance of predicting ICB therapeutic response based on HPRS in the IMvigor210 cohort, consisting of 299 samples. Kaplan–Meier analysis showed that high HPRS had worse survival than low HPRS, although the *p*-value was 0.06, and this might be due to the usage of BCR-free survival when formulating HPRS and the usage of overall survival (OS) in this dataset (Figure 8A). We then found a significant difference in hypoxia scores between the immunotherapy response and non-response (Figure 8B). To assess the effect of HPRS in predicting ICB therapeutic response, we used the SVM algorithm to construct a model. The best effect was obtained when the kernel function was “radial.” We trained the model on the training set, and this model also achieved a good accuracy (0.7879) on the test set, indicating that HPRS is a good predictor of ICB therapeutic response.

## Discussion

The precise definition of hypoxia is a pathological phenomenon that occurs when the body tissue cannot get enough oxygen or cannot use oxygen. Hypoxic microenvironment can lead to upregulation of HIF-1α expression in tumor cells, which in turn activates downstream target genes and promotes metabolic reprogramming, epithelial–mesenchymal transition, invasion, metastasis, cancer stem cell maintenance, immune evasion, and resistance to chemotherapy and radiation therapy (Schito and Semenza, 2016; Farina et al., 2020). To date, some hypoxia gene signatures for prognosis have been developed in different cancer types, such as clear cell renal cell carcinoma (Gui et al., 2021), bladder cancer (Cao et al., 2021), lung adenocarcinoma (Sun et al., 2020), breast cancer (Gong et al., 2020), and hepatocellular carcinoma (Mo et al., 2021). Prostate cancer has been shown to be a tumor characterized by hypoxia (Bhandari et al., 2019). Therefore, in this study, we focused on finding hypoxia-related genes and exploring whether they could be used as prognostic markers.

The WGCNA algorithm, designed to study the relationship between genes and sample phenotype, can be used to identify complex mechanisms responsible for target phenotypes. Through the preliminary screening of WGCNA, we obtained a set of hypoxia-related genes, which were significantly enriched in cancer-related pathways, demonstrating the reliability of our method. In the end, we obtained a signature consisting of 22 genes for predicting prognosis. Whether in the training dataset or in several other independent datasets, HPRS showed good performance in predicting prognosis.

GSEA results of different hypoxia levels, KEGG enrichment results of 22 gene signatures, and GSEA results of different HPRS groups, all suggested that phagosomes might play an important role in tumor hypoxia. In addition, the high-HPRS group was also associated with many pathways related to tumors, which proves that high hypoxia levels can promote tumor metastasis. We hope to verify this in subsequent studies. However, the low-HPRS group was mainly related to metabolism-related pathways, which means that there is a dynamic metabolic feature. The immune micro-environment plays a critical role in the tumorigenesis and progression. CIBERSORT, in our study, demonstrated that hypoxia might act on multiple T cells, activated NK cells, and M1 macrophages by various ways, suggesting that hypoxia might exert its anti-tumor effects by suppressing T cells and NK cells.

We observed that high HPRS was significantly correlated with more aggressive molecular changes such as TP53 mutation and amplifications of driver oncogenes. During the PC progression, these genomic alterations drive rapid proliferation rates by depleting oxygen and producing abnormal vasculature. The mutation of TP53 has an effect on cancers evading targeted therapies through a mechanism known as lineage plasticity (Mu et al., 2017).

In order to escape the pursuit of T cells, tumor cells also produce some inhibitory signals on their own surface, thereby inhibiting the immune function of T cells through immune checkpoints. ICB therapy such as PD-1 antibody can significantly improve the survival of patients with various tumors by blocking these inhibitory signals, but only some patients respond. Finding a good marker to predict response to ICB therapy is critically essential for patients. As the aforementioned CIBERSORT result demonstrated that hypoxia might act on multiple T cells and activated NK cells, we are more convinced that hypoxia-related genes have a good effect in predicting ICB therapeutic response. Our SVM model also proved that HPRS had a good performance on predicting the therapeutic response.

Our research also inevitably has some limitations. Our analysis data are derived from tumor tissue as a whole, but tumor tissue contains not only cancer cells but also other non-cancer cells such as immune cells. The multiple datasets used are data from different platforms, which makes these data naturally different. As there is no ICB therapy cohort for PC, we used the largest cohort consisting of 299 urothelial carcinoma samples for analysis.

## Conclusion

Our study illustrates the crucial role of hypoxia in PC. Hypoxia gene-related prognostic model has been established and found to have good performance. HPRS could be a useful tool to select patients who may benefit from ICB therapy and thus to facilitate personalized management of PC.

## Data Availability

The datasets used and analyzed during the current study are available from the corresponding author upon reasonable request.

## Abbreviations

Androgen receptor = AR; androgen deprivation therapy = ADT; area under the ROC curve = AUC; biochemical recurrence = BCR; cell adhesion molecules = CAMs; false discovery rate = FDR; Gene Expression Profiling Interactive Analysis = GEPIA; Gene Expression Omnibus = GEO; hypoxia-related prognostic risk score = HPRS; immune checkpoint blockade = ICB; Kaplan–Meier = KM; Kyoto Encyclopedia of Genes and Genomes = KEGG; overall survival = OS; prostate cancer = PC; prostate-specific antigen = PSA; receiver operating characteristic = ROC; robust multichip average = RMA; support vector machines = SVMs; single-sample gene set enrichment analysis = ssGSEA; The Cancer Genome Atlas = TCGA; transcripts per million = TPM; weighted gene co-expression network analysis = WGCNA.

## References

[B1] Barsoum I. B. Smallwood C. A. Siemens D. R. Graham C. H. (2014). A mechanism of hypoxia-mediated escape from adaptive immunity in cancer cells. Cancer Res. 74 (3), 665–674. 10.1158/0008-5472.CAN-13-0992 24336068

[B2] Bhandari V. Hoey C. Liu L. Y. Lalonde E. Ray J. Livingstone J. (2019). Molecular landmarks of tumor hypoxia across cancer types. Nat. Genet. 51 (2), 308–318. 10.1038/s41588-018-0318-2 30643250

[B3] Bu D. Luo H. Huo P. Wang Z. Zhang S. He Z. (2021). KOBAS-I: Intelligent prioritization and exploratory visualization of biological functions for gene enrichment analysis. Nucleic Acids Res. 49 (W1), W317–W325. 10.1093/nar/gkab447 34086934 PMC8265193

[B4] Cao R. Ma B. Wang G. Xiong Y. Tian Y. Yuan L. (2021). Characterization of hypoxia response patterns identified prognosis and immunotherapy response in bladder cancer. Mol. Ther. Oncolytics 22, 277–293. 10.1016/j.omto.2021.06.011 34553019 PMC8426180

[B5] Chae Y. C. Vaira V. Caino M. C. Tang H. Y. Seo J. H. Kossenkov A. V. (2016). Mitochondrial akt regulation of hypoxic tumor reprogramming. Cancer Cell. 30 (2), 257–272. 10.1016/j.ccell.2016.07.004 27505672 PMC5131882

[B6] Colaprico A. Silva T. C. Olsen C. Garofano L. Cava C. Garolini D. (2016). TCGAbiolinks: An R/bioconductor package for integrative analysis of TCGA data. Nucleic Acids Res. 44 (8), e71. 10.1093/nar/gkv1507 26704973 PMC4856967

[B7] Crawford E. D. Schellhammer P. F. McLeod D. G. Moul J. W. Higano C. S. Shore N. (2018). Androgen receptor targeted treatments of prostate cancer: 35 Years of progress with antiandrogens. J. Urol. 200 (5), 956–966. 10.1016/j.juro.2018.04.083 29730201

[B8] Farina A. R. Cappabianca L. Sebastiano M. Zelli V. Guadagni S. Mackay A. R. (2020). Hypoxia-induced alternative splicing: The 11th hallmark of cancer. J. Exp. Clin. Cancer Res. 39 (1), 110. 10.1186/s13046-020-01616-9 32536347 PMC7294618

[B9] Gong P. J. Shao Y. C. Huang S. R. Zeng Y. F. Yuan X. N. Xu J. J. (2020). Corrigendum: Hypoxia-Associated prognostic markers and competing endogenous RNA Co-expression networks in breast cancer.. Front. Oncol. 10, 637481. 10.3389/fonc.2020.637481 33585259 PMC7880053

[B10] Gui C. P. Wei J. H. Chen Y. H. Fu L. M. Tang Y. M. Cao J. Z. (2021). A new thinking: Extended application of genomic selection to screen multiomics data for development of novel hypoxia-immune biomarkers and target therapy of clear cell renal cell carcinoma. Brief. Bioinform. 22 (6), bbab173. 10.1093/bib/bbab173 34237133

[B11] Hänzelmann S. Castelo R. Guinney J. (2013). Gsva: Gene set variation analysis for microarray and RNA-seq data. BMC Bioinforma. 14, 7. 10.1186/1471-2105-14-7 PMC3618321 23323831

[B12] Irizarry R. A. Hobbs B. Collin F. Beazer-Barclay Y. D. Antonellis K. J. Scherf U. (2003). Exploration, normalization, and summaries of high density oligonucleotide array probe level data. Biostat. Oxf. Engl. 4 (2), 249–264. 10.1093/biostatistics/4.2.249 12925520

[B13] Jain S. Lyons C. A. Walker S. M. McQuaid S. Hynes S. O. Mitchell D. M. (2018). Validation of a Metastatic Assay using biopsies to improve risk stratification in patients with prostate cancer treated with radical radiation therapy. Ann. Oncol. 29 (1), 215–222. 10.1093/annonc/mdx637 29045551 PMC5834121

[B14] Langfelder P. Horvath S. (2012). Fast R functions for robust correlations and hierarchical clustering. J. Stat. Softw. 46 (11). 10.18637/jss.v046.i11 PMC3465711 23050260

[B15] Lin W. Wu S. Chen X. Ye Y. Weng Y. Pan Y. (2020). Characterization of hypoxia signature to evaluate the tumor immune microenvironment and predict prognosis in glioma groups. Front. Oncol. 10, 796. 10.3389/fonc.2020.00796 32500034 PMC7243125

[B16] Mayakonda A. Lin D. C. Assenov Y. Plass C. Koeffler H. P. (2018). Maftools: Efficient and comprehensive analysis of somatic variants in cancer. Genome Res. 28 (11), 1747–1756. 10.1101/gr.239244.118 30341162 PMC6211645

[B17] Mariathasan S. Turley S. J. Nickles D. Castiglioni A. Yuen K. Wang Y. (2018). TGFβ attenuates tumour response to PD-L1 blockade by contributing to exclusion of T cells. Nature 554 (7693), 544–548. 10.1038/nature25501 29443960 PMC6028240

[B18] Mo Z. Liu D. Rong D. Zhang S. (2021). Hypoxic characteristic in the immunosuppressive microenvironment of hepatocellular carcinoma. Front. Immunol. 12, 611058. 10.3389/fimmu.2021.611058 33679749 PMC7928397

[B19] Mortensen M. M. Høyer S. Lynnerup A. S. Ørntoft T. F. Sørensen K. D. Borre M. (2015). Expression profiling of prostate cancer tissue delineates genes associated with recurrence after prostatectomy. Sci. Rep. 5, 16018. 10.1038/srep16018 26522007 PMC4629186

[B20] Mu P. Zhang Z. Benelli M. Karthaus W. R. Hoover E. Chen C. C. (2017). SOX2 promotes lineage plasticity and antiandrogen resistance in TP53- and RB1-deficient prostate cancer. Sci. (New York, NY) 355 (6320), 84–88. 10.1126/science.aah4307 PMC5247742 28059768

[B21] Newman A. M. Steen C. B. Liu C. L. Gentles A. J. Chaudhuri A. A. Scherer F. (2019). Determining cell type abundance and expression from bulk tissues with digital cytometry. Nat. Biotechnol. 37 (7), 773–782. 10.1038/s41587-019-0114-2 31061481 PMC6610714

[B22] Ross-Adams H. Lamb A. D. Dunning M. J. Halim S. Lindberg J. Massie C. M. (2015). Integration of copy number and transcriptomics provides risk stratification in prostate cancer: A discovery and validation cohort study. EBioMedicine 2 (9), 1133–1144. 10.1016/j.ebiom.2015.07.017 26501111 PMC4588396

[B23] Scharping N. E. Rivadeneira D. B. Menk A. V. Vignali P. D. A. Ford B. R. Rittenhouse N. L. (2021). Mitochondrial stress induced by continuous stimulation under hypoxia rapidly drives T cell exhaustion. Nat. Immunol. 22 (2), 205–215. 10.1038/s41590-020-00834-9 33398183 PMC7971090

[B24] Schito L. Semenza G. L. (2016). Hypoxia-inducible factors: Master regulators of cancer progression. Trends Cancer 2 (12), 758–770. 10.1016/j.trecan.2016.10.016 28741521

[B25] Sun J. Zhao T. Zhao D. Qi X. Bao X. Shi R. (2020). Development and validation of a hypoxia-related gene signature to predict overall survival in early-stage lung adenocarcinoma patients. Ther. Adv. Med. Oncol. 12, 1758835920937904. 10.1177/1758835920937904 32655701 PMC7333486

[B26] Sung H. Ferlay J. Siegel R. L. Laversanne M. Soerjomataram I. Jemal A. (2021). Global cancer statistics 2020: GLOBOCAN estimates of incidence and mortality worldwide for 36 cancers in 185 countries. Ca. Cancer J. Clin. 71 (3), 209–249. 10.3322/caac.21660 33538338

[B27] Tan M. H. Li J. Xu H. E. Melcher K. Yong E. L. (2015). Androgen receptor: Structure, role in prostate cancer and drug discovery. Acta Pharmacol. Sin. 36 (1), 3–23. 10.1038/aps.2014.18 24909511 PMC4571323

